# Bacteriological Monitoring and Sustainable Management of Beach Water Quality in Malaysia: Problems and Prospects

**DOI:** 10.5539/gjhs.v4n3p126

**Published:** 2012-05-01

**Authors:** Ayokunle Christopher Dada, Ahmad Asmat, Usup Gires, Lee Yook Heng, Bandele Oluwaseun Deborah

**Affiliations:** 1 School of Biosciences & Biotechnology, Faculty of Science & Technology, Universiti Kebangsaan Malaysia, 43600 UKM Bangi, Selangor, Malaysia; 2 School of Environmental & Natural Resource Sciences, Faculty of Science & Technology, University Kebangsaan Malaysia, 43600 UKM Bangi, Selangor, Malaysia; 3 School of Chemical Sciences & Food Technology, Faculty of Science & Technology, University Kebangsaan Malaysia, 43600 UKM Bangi, Selangor, Malaysia; 4 Institute of Bioscience, Universiti Putra Malaysia, 43400 UPM Serdang, Selangor, Malaysia

**Keywords:** beach quality, bacteriological monitoring, sustainable management, Malaysia

## Abstract

Despite the growing demand of tourism in Malaysia, there are no resolute efforts to develop beaches as tourist destinations. With no incentives to monitor public beaches or to use them in a sustainable manner, they might eventually degenerate in quality as a result of influx of pollutants. This calls for concerted action plans with a view to promoting their sustainable use. The success of such plans is inevitably anchored on the availability of robust quality monitoring schemes. Although significant efforts have been channelled to collation and public disclosure of bacteriological quality data of rivers, beach water monitoring appears left out. This partly explains the dearth of published information related to beach water quality data. As part of an on-going nation-wide surveillance study on the bacteriological quality of recreational beaches, this paper draws on a situation analysis with a view to proffering recommendations that could be adapted for ensuring better beach water quality in Malaysia.

## 1. Introduction

Beaches are characteristic sites for human recreation. Gaining patronage from both local and international tourists, arguably, beaches underpin economic development as they support commercial developments and tourism in coastal areas (Klein et al., 2004). Given the intense demand, beaches are under threat worldwide from a variety of human pressures including coastal pollution from industrial and anthropogenic courses in addition to the effects of global climate change (Schlacher et al., 2006). Added to this pressure is the increasingly rapid growth of coastal populations, coupled with increased availability of leisure time (De Ruyck et al., 1997; Caffyn and Jobbins, 2003; Fanini et al., 2006; Schlacher et al., 2007). For instance, using 1995 estimates and a 100 km coastal buffer with a 10 km ’safe area’ falling into the sea, geographic information technologies puts at 98% the number of people residing within Malaysia’s 9,323 km coastal stretch (EarthTrends 2003). The increasing pressure from rapid urbanisation and economic development thus present significant challenges for coastal management strategies aimed at sustainable tourism. Added to the existing stress level is the increasing growth of tourism in the nation.

Malaysia remains one of the top notches when it comes to tourism in Asia. Recent years have seen the Asia Pacific shares in world tourist arrivals increasing from 1.3% in 1960 to more than 20% in 2005 (Sivalingam, 2007). The government of Malaysia has actively promoted tourism as an important industry by providing infrastructure, fiscal incentives, subsidies, and public funding for projects that enhance the image of the country and tourist destinations within the country (Sivalingam, 2007). Contributing up to 8% of the GDP of Malaysia, tourism has remained an important source of foreign exchange earnings in Malaysia especially after the collapse of primary commodity prices in the 1980s. The government of Malaysia also recognises tourism as a National Key Economic Area. For instance, eighteen million tourists visited Malaysia in 2006 and spent an estimated RM36 billion (IEM, 2011). From 2006 to 2010, Malaysia recorded an estimated 23.6 million tourist arrivals with receipts of RM53.4 billion and an increased tourist per capita expenditure to over RM 2,000 (EPU, 2010). Increased tourism may imply increased demand for beach-based recreation in Malaysia. However, efforts to develop beaches as tourist destinations appear lacking going by its non-inclusion of beach-related tourism in the Ninth and Tenth Malaysia Plan. These Plans contains the aspirations of the Government Transformation Programme, new policy directions and strategies as it charts the developmental plans of the nation for 2006-2010 and 2011-2015 respectively. Identifiable in both is the omission of plans to promote the sustainable management of coastal recreational beaches.

Sustainable coastal beach management offers rewarding promises. It will however involve a multidisciplinary and iterative process, within a national framework, that will be established to cover the full cycle of information collection, planning, decision making, management and monitoring of its implementation. Starting up with a dependable information collection system is inevitably anchored on the availability of robust quality monitoring schemes. Bacteriological monitoring of beaches is necessary to protect public health and to promote sustainable use of the beaches. Following a general paraphrase of the theme of the paper in this section, the second section presents with particular emphasis on bacteriological components, a literature review of information on beach water quality in Malaysia that have been published till date. The third section cuts across the various stakeholders involved, highlighting core challenging issues associated with the quality of coastal beaches in Malaysia. Prospecting towards a better state of coastal beaches; the fourth section proffers recommendations that could ultimately create an atmosphere which balances environmental, economic and recreational objectives within the limits set by natural dynamics. The fifth section concludes.

## 2. Bacteriological Quality of Coastal Beaches in Malaysia

Faecal indicator bacteria (FIB) are widely accepted and used in water quality studies to assess the level of fecal contamination in water bodies (Garrido-Perez et al. 2008). The presence of these organisms has also been used to estimate the potential human health risks of other pathogenic organisms of fecal origin. Constant monitoring of recreational waters for faecal indicator organisms is necessary to reduce bathers’ exposure to pathogens and to ultimately protect public health. To have a clear grasp of information available in the public domain on the bacteriological quality of coastal beaches in Malaysia, scholarly search engines were used. Queries were run on PubMed, ScienceDirect and GoogleScholar sites using a combination of one or more of the following subject headings: coastal, beach(es), microbiological, bacteriological, quality, characterization, antibiotic, resistance, marine, sea water and Malaysia. MEDLINE/PubMed Journal Literature provides access to over 12 million references from 4600 biomedical journals. SciVerse ScienceDirect scientific database contains more than 10 million journal articles and book chapters. Google scholar provides a search of scholarly literature across many disciplines and sources, including theses, books, abstracts and articles. Additional publications were identified by reviewing references in selected articles. The queries were also run on the general google site for all-inclusive search results. Where necessary, individual authors were contacted for electronic reprints of full papers in instances where the abstract alone was available for viewing online. A limitation to this approach however, was that no formal attempt was made to identify unpublished studies. Physical visits were made to some of the beaches for which information has been published.

Based on the search, a number of internet blogs presented certain viewpoints on the quality of popular recreational beaches in Malaysia. These claims are however subjective and laden with a bias for aesthetic quality. Agreeably, translating such subjective viewpoints on the bacteriological state of recreational beaches for high-level policy decision making is far from an achievable reality. No results were available for Malaysia-specific studies on antibiotic resistance in recreational waters. To the best of our knowledge therefore, no single study has aimed at the occurrence of bacteria antibiotic resistance among recreational beaches despite the globally recognised health concern that the subject raises. The search on general bacteriological studies on recreational marine waters yielded 3 scientific articles and one institution-based non-journal articles. In terms of frequency and intensity of sampling, the Department of Environment in collaboration with the Water Environment Partnership in Asia published report of Marine water quality in Malaysia. In 2006, a total of 1,035 marine water samples from 229 monitoring stations were analyzed. Furthermore, 344 samples were also collected and analyzed from islands, mainly development islands, resort islands, marine park islands and protected islands. Public beaches apparently were not included in the sampling regimes. Perhaps the first report in this direction, Law and Othman (1985) reported the distribution of fecal coliform bacteria at the popular Port Dickson recreational beach. Five years later, the same authors (Law and Othman, 1990) reported sewage pollution at the same location in a study undertaken to monitor the distribution of faecal coliform bacteria following measures taken by relevant authorities to minimise levels of sewage pollution then. In this study, an extensive sampling was done that covered the entire PD coast and extended in graduated distances to a maximum of 5km towards the sea. Samples were collected eight times in 1987 and 1989 for each of the sixteen sampling stations considered in the study. Hamzah et al (2011), in a most recent report, published data collated from a five-time sampling regime per station for Saujana, Kemang Indah, Blue Lagoon and Teluk Kemang public beach between October 2000 to March 2001. No study has attempted to focus on the implication of bathers population density on bacteriological quality via comparisons of samples collected during weekdays or weekends. Thus, it might be that current monitoring practices do not capture levels of FIBs during peak swim time at public beaches, these which normally fall on weekends or public holidays in Malaysia. This possibility was also reported by Sgambati (2009).

For most of the studies considered in this review, the choice of bacteriological parameters used was largely conventional faecal indicator bacteria. Lee et al. (2009) reported a study on characterization of culturable bacteria from selected coastal environments along the Straits of Malacca. Using an approach based on 16S rDNA restriction fragment length polymorphism (RFLP), and 16S rDNA sequence analysis, the study determined the bacterial diversity in these coastal waters presenting a comparison analysis of culturable versus unculturable bacteria. This study however was not considered in this review because it did not set out to determine the quality of recreational coastal waters. Law and Othman (1990) employed the use of faecal coliforms (*E. coli*) counts as measure of the level of faecal pollution. Two decades after this report was published, Hamzah et. al. (2011) published findings on another approach that employed a combination of total coliforms, faecal coliforms (*E. coli*) and faecal streptococci. In the report, statistically tested comparisons of FIB counts were made for the purpose of selecting the ideal indicator for marine water pollution monitoring. In scientific literature, the use of coliphages has been suggested by some quarters. Of all the reviewed publications, only one (Hamzah et. al., 2011) attempted this approach alongside the use of conventional FIBs for coastal water quality monitoring. The absence of studies using this alternative approach may be arguably due to the absence of specific coliphage guidelines. The DOE employs E. coli as an indicator of marine pollution (DOE 2006). Most of the reported studies reviewed employed benchmarking against specific guidelines in reporting bacteriological quality data. Law and Othman (1990) used the DOE-UM (1986) interim standard for Malaysian recreational water and the WHO (1975, 1977b) guidelines for bathing and shellfish harvesting waters. The Interim Marine Water Quality standards (IMWQS) have been used as the benchmark for the marine monitoring program in 1978 for Peninsular Malaysia and in 1985 for Sabah and Sarawak. Hamzah et al. (2011) also cited the 100 MPN/100 mL limit for recreational use with body contact set by the Department of Environmental Malaysia (DOE 2006) in addition to the 35 cfu/100 mL limit for fecal streptococci set for recreational water by U.S. EPA (1986). Furthermore, coliphage counts observed during the study were compared with the coliphage level Ph_80_= 300 pfu/100 mL and Ph_95_= 2000 pfu/100 mL as suggested by Dionisio et al. (2002) for bathing waters.

The results and study conclusions of all reviewed reports were somewhat similar, all referring to varying levels of pollution in the considered study locations. In an instance, Law and Othman (1990) reported consistently high faecal coliform counts (ranging from 2 x 10^4^ MPN/100mL to 2 x 10^6^ MPN/100mL) for a sampling station at the harbour receiving waste water discharged from the town. Similarly high counts were observed for two other stations situated in close proximity to the area receiving waste water discharge. At another sampling location dominated with the presence of hotels and condominiums along the PD coast, the faecal coliform count counts obtained were remarkably high but not as high as obtained for the area receiving waste water discharges from the town. No statistical comparisons were however made by the study. Calling for more stringent standard for treatment and discharge of sewage effluents into near shore waters, Law and Othman (1990) concluded that efforts to clean up the Port Dickson coastal area from sewage pollution seemed to show no significant improvement. In a previous study by the same authors (Law & Othman, 1985), very low counts of FIBs were observed in some locations that could be considered safe for recreational purposes. Calling for proper sewage disposal management, Law and Othman (1985) however warned that with rapid housing development in these areas, it will not be long before these beaches become polluted by sewage. This eventually proved true as reported five years later (Law & Othman, 1990)

A study done by Kadaruddin (1997) also reported the mean faecal coliform counts of water samples from Teluk Kemang and Blue Lagoon were 1950 MPN/100 mL and 141 MPN/100 mL respectively. Both values exceeded the IMWQS standards. In another study (Hamzah et al., 2011), total coliform and faecal coliform counts ranged from 2.5 x 10^2^ to 1.5 x 10^6^ cfu/100ml and 1.5 x 10^2^ to 2 x 10^4^ cfu/mL respectively. Hamzah et al. (2011) also reported higher faecal streptococci counts ranging from 1.5 x 10^3^ to 2.5 x 10^6^ cfu/100mL. Furthermore, for all sampling points considered in the study, there was a positive and strong correlation between total coliform and fecal coliform (R-value, 0.75) but a weak correlation between faecal coliform and faecal streptococci (R-value, 0.41) and a negative correlation between faecal coliform and coliphages. Worthy of note is the argument presented by Hamzah et al. (2011) on the effect of sunlight on the survival of faecal coliforms and streptococci in sea water. In Malaysia, a tropical country with all year round sunlight, the probable effects of sunlight on these FIBs are important considerations yet to be reported. Based on comparisons with available guidelines, Hamzah et al. (2011) concluded that seawater at the studied location is not safe for recreational purposes confirming an earlier submission by Law and Othman (1985).

## 3. State of Coastal Beaches in Malaysia: Problems

A major problem identifiable as contributing factor to the current state of coastal beaches in Malaysia is that of an absence of political will power to set in motion action plans with a view to improving the quality of these beaches. For example, Sivalingam (2007) opines that the government has no plans to clean the beaches along the Straits of Malacca, the Johore Straits and the South China Sea. Yet public beaches in those areas are still popular among tourists. There are also no early warning systems to disclose to bathers the risk of pathogen exposure in these recreational waters. Although significant efforts have been made by the government in terms of collation and public disclosure of data related to bacteriological quality of rivers, beach water monitoring appears left out. This understandably may be because more than 90% of Malaysia’s water supply comes from rivers and streams (EPU, 2010). Thus, efforts to tackle river pollution have always been, and will continue to be given due emphasis. With no formal surveillance scheme in place, property developers within close proximity to the beaches might abuse the situation leaving the vulnerable public who visit these beaches at potential health risk.

It is often argued that apparent negligence on the part of the government has heralded form of adaptation, in favour of the wealthy. The recent years has seen private hoteliers taking advantage of the situation as they capture property rights over the sea and encroach even farther into the sea, without any institutional framework put in place to monitor their activities. This however leads to another potentially debatable topic as beaches transcend from being a public good to a private commodity meant only for those that can afford to pay. Social exclusion in coastal tourism is a well known phenomenon. Even in developed nations, the continuing controversies over public access to beaches linger on. For instance, according to the Florida State’s Department of Environmental Protection, at least 60 percent of Florida beaches are private, offering little or no public access (Costello, 2005; Kranz, 2009). This is because over the last three decades, local governments have routinely ceded public access points to developers, allowing waterfront communities to exclude others from the dry sand beach in exchange for the significant tax revenues these communities provide (Sullivan, 2003). In Malaysia, given their power of investments, these private island resorts and hotels built on the sea are usually guarded preventing access to members of the public who are not willing to pay. In such situation, coastal bathing water becomes a private good rather than the public good it is meant to be as it delineates two social categories; the paying public versus general public.

On the other hand, excludability gives the service provider (the seller i.e. hotel management) the chance to make a profit from producing and selling the product. Thus attempts to yield returns on investments by the hoteliers is quite logical as they charge a fee for their services. However, the potential of oppressing the poor brings in a hitherto ignored form of hegemony. Some of these hotels invest heavily on enclosed portions of the seawater, termed private swimming pools, which are protected and maintained in good quality as a result of regular treatment. These portions are used only by individuals paying at the hotel. On the other hand, wastes from these private hotels end up being discharged into the receiving seawater which is available for the general public i.e. the unguarded section referred to as ‘public beach’. This brings in the issue of rivalry where one person’s consumption of a product (beach) reduces the amount (quality) left for others to consume and benefit from. Physical visits to some of these coastal beach hotels reveal that only thin lines of demarcation exist between the ‘private’ beach area and the ‘public’. Private hotels that discharge waste into the sea facilitate an environment where the rich generate externalities for the poor to pay.

Just a few meters away where beaches are not guarded by hotel security details, the free-rider problem of pollution prevails as described by Pasour (1981). With no incentives to monitor and protect these public beaches or to use them in a sustainable manner, they might ultimately degenerate in quality as a result of influx of pollutants from the beach users. With no adequate awareness programs aimed at beach protection among the general citizenry, the public may also contribute to pollution of the beaches. Beaches in Malaysia are also under the threat of direct sewage discharge pipes on the coast. An example is the sewage drain outfall just northwards of the central bathing area at the popular Port Dickson public beach. Apart from this, there are several other wastewater pipe lines from nearby houses that discharge wastewater directly into the beach (Kadaruddin 1997). The implication of this is that intertidal sands (Ulfig et al., 1997; Salvo and Fabiano, 2007) and surf-zone waters (Bonilla et al., 2006, 2007; Noble et al., 2006) end up being contaminated by pathogens, which are delivered to the sea by sewage systems discharging directly into coastal waters (Mardon and Stretch, 2004; Araujo and Costa, 2007). Bacterial levels in bathing waters that exceed human health standards usually trigger beach closures and public warnings in many developed nations. In Malaysia, such early warning systems established with a mandate to inform bathers of water conditions are lacking. Availability of appropriate waste-water management practices, the timing and intensity of local rainfall events and subsequent runoff and the strength of mixing and dispersion in the surf zone (Stretch & Mardon, 2005) remain major determinants of the quality of any given beach. Apart from reducing the quality of beach water, pollution makes beaches unattractive and unsafe as tourists’ destinations. There is therefore the need for concerted action plans with a view to promoting the sustainable use and protection of these beaches. The success of such is inevitably anchored on the availability of robust quality monitoring schemes, most of which are lacking for these beaches.

**Figure 1:**
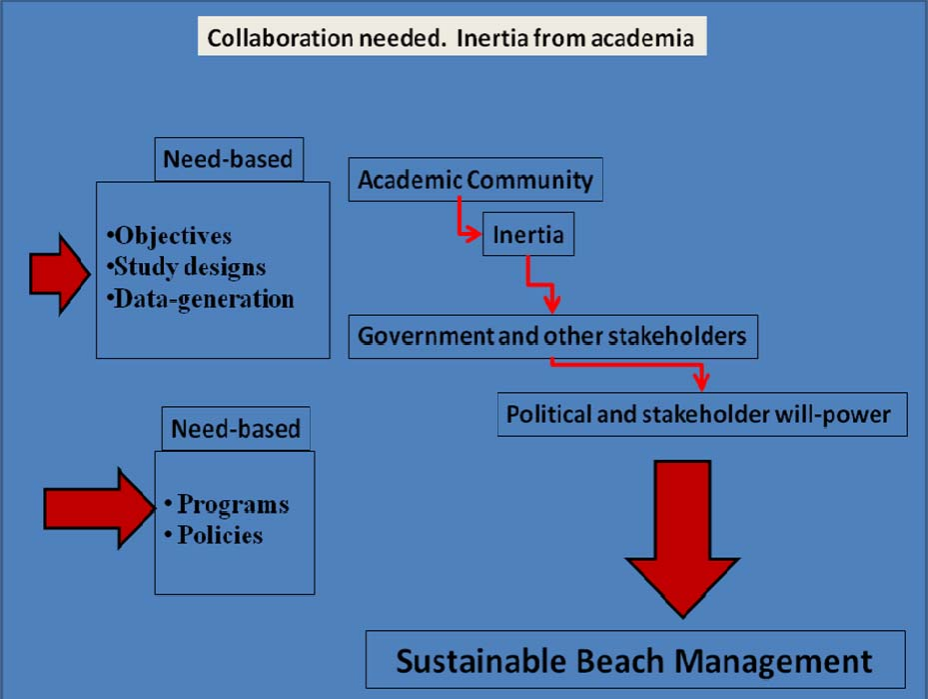
Inertia from academic community towards sustainable beach management

Having known that constant monitoring of recreational waters for faecal indicator organisms is necessary to protect public health, the onus lies on the academic community to work together with the government, civil society groups and other relevant stakeholders to make this a reality. The research community could help bridge the gap by generating scientific data based on carefully planned and funded experimental designs, for the purpose of supporting early warning systems and triggering public behavioural change and the political will power for high-level policy decision making on sustainable beach management (Figure 1). However, the attention of the academia seems far from this direction, evident in the lack of sufficient published information on the quality of coastal beach waters used for recreational purposes. It has been argued however in some quarters that the entirety of environment issues as it is being aggressively promoted might be a western plot to impoverish the government of developing nations. This may explain the reluctance of indigenous scientists to devote resources into such surveillance studies.

Arguably, the prominent negligence by indigenous scientists on studies that focus on bacteriological quality monitoring of beaches, may also be due to the national drive for a tenacious focus on biotechnology as Malaysia prioritizes her Vision 2020 ambition. The ninth Malaysia Plan emphasizes this. In the 10^th^ Malaysian Plan, a somewhat similar trend is observable as it does not include environmental biotechnology neither does it list water resources sector as a key sector for research development (Azhar, 2000). Beaches were only mentioned twice in the most recent 451-page document, in the first instance, under a framework for restructuring Solid Waste Management for the upkeep of communal facilities, such as markets and beaches (EPU, 2010: pp 288). Classifying beaches and markets as being ‘communal facility’ apparently presents them as something designed, built, installed, etc., to serve a specific function affording a convenience or service used or shared in common by everyone rather than a natural resource to be protected. Within this Waste Management framework, specific action plans were stated for issues related to sanitation for residential and commercial premises such as waste handling process optimization and then a general statement on ‘improved environment for shared infrastructure such as drains, roads, markets and beaches’. No specific action plans for sustainable use, management or monitoring of recreational beaches were included.

Also, there appears to be a problem with inter-agency coordination especially between the central government, state governments, municipalities and town councils which makes it difficult to identify who is responsible for recreation-based beach water monitoring, protection and management. Azhar (2000) also argues that there is no single agency entrusted with the overall responsibility of holistic water planning and management in the country. Table 1 depicts these conflicting functional responsibilities of water sector agencies. Although general water quality data collection is delegated to the Department of Environment, recreation is zoned under Department of Irrigation and Drainage, Town and Country planning and Local authorities. It is hoped that with Solid Waste Management (SWM) and Public Cleansing (PC) responsibilities transferred to the federal government as stated in the 10th Malaysia Plan (EPU, 2010: pp 289), local authorities will be more empowered to focus on core functions such as monitoring, licensing, enforcement, planning and development especially as it relates to recreational beaches.

**Table 1:** Functional responsibilities of water sector agencies in Malaysia

FUNCTION	Water supply	Water sanitation	Irrigation	Hydropower	Flood control	Water quantity regulation	Water quality regulation	Watershed management	Integrated area development	Data collection	Research	Cloud seeding	Ports and navigation	Fisheries	Recreation
**AGENCY**															
Department of Chemistry															
Department of Environment															
Department of Fisheries															
Department of Forestry															
Department of Irrigation and Drainage															
Department of Mineral and Geoscience															
Department of Sewerage Services															
Department of Town and Country Planning															
Local Authorities															
Malaysian Meteorological Services															
Ministry of Health (Engineering Division)															
Ministry of Transport															
National Hydraulic Research Institute															
National Water Resources Council															
Royal Malaysian Air Force															
State Water Resources Management Authority															
Tenaga National Berhad															
Waterworks Department															

Source: Azhar, 2000

It is worthy of note that the few published papers on beach quality monitoring merely adopted traditional methodologies. These rudimentary approaches are often biased and time consuming. Also, traditional methods of bacterial enumeration are often insufficient for monitoring the specific microbes critical for important biochemical reactions in complex, mixed microbial communities (Schneegurt and Kulpa, 2010). This opens up opportunities for research into potentials of biotechnology for environmental monitoring. A necessary precondition for environment-friendly processing is the development of highly specific and efficient monitoring devices (Zechendorf, 2009). Several new methodologies, for example, based on DNA/RNA analyses exist which can detect and quantify phylogenetic groups on the basis of rDNA sequences and relevant structural genes. These include those based on sequence analysis, %G+C content (percentage of the nucleotides, guanine and cytosine), PCR (Polymerase Chain Reaction) based methods, DGGE (Denaturing Gradient Gel Electrophoresis) and TGGE (Temperature Gradient Gel Electrophoresis) (FWR, 2000). However, most are these are relatively new, bulky and currently too expensive to be considered for routine analysis in the short to medium term.

Another well documented disadvantage of available biosensors is that they exhibit high specificity, lack of stability and short life-time (Griffiths and Hall, 1993). Although the issue of specificity presently makes them unsuitable for mass marketing, the need for indigenous solutions within this broader framework is important. Despite their promising long term potential for investigative or diagnostic studies on the environment, indigenous biotechnology projects that focus on their adaptation into simple, hand-held or remote application biosensor formats for detecting, identifying, and enumerating water- borne microbial pathogens are relatively few. In the past five years, several national initiatives were launched with investments in new growth areas reflecting the trend towards more capital-intensive, high value-added and high technology projects. For the period 2005-2009, most of the investments on biotechnology were in a range of activities including genomic science, stem cells, biodiesel and medical devices involving predominantly agriculture, industry and healthcare. Environmental Biotechnology despite the promise it offers for sustainable resource management appear left out. Given this national direction, researchers may be ultimately interested in projects that will secure funding, based on the priority of the government of the day, and not necessarily those aim at environmental sustainability via improved surveillance and environmental protection.

## 4. Prospecting towards a Better State of Coastal Beaches in Malaysia

Beaches are important coastal resources. Globally, countries that promote their coastal areas for tourism are increasingly becoming aware of the need to monitor and protect these areas in order to maintain their natural beauty and help ensure their long-term vitality as tourism destinations. It is desired that Malaysia is not left behind in this move towards achieving sustainable resource management. According to a definition by the International Finance Corporation, sustainable resource management is the use, development and protection of resources in away, or at a rate, which enables people and communities to provide for their present social, economic and cultural well-being while also sustaining the potential of those resources to meet the reasonable foreseeable needs of future generations and safeguarding the life-supporting capacity of air, water and soil ecosystem (UDC, 2011). In other words, managing complex systems, such as coastal areas, requires an integrated approach capable of coordinating the implementation of all three major objectives of sustainable development (environmental, social and economic). This includes bringing together the multiple, interwoven, overlapping interests in the coastal area in a coordinated and rational manner, harnessing coastal resources for optimum social and economic benefit for present and future generations without prejudicing the resource base itself, while maintaining ecological processes (UNEP, 2009). Particularly for coastal beaches, another school of thought suggests an approach termed Integrated Coastal Zone Management (ICZM); a continuous, proactive and adaptive process of resource management for sustainable development in coastal areas (Cicin-Sain & Knecht, 1998b). Whatever the approach, the message is clear – sustainability. A proffered direction towards achieving a sustainable state of beaches in Malaysia suggestively begins with a re-aligning of priorities. In either approaches increasingly recognised is the fundamental role science or environmental monitoring can play as they are vital tools in environmental impact assessments (Clark, 1992). One of such approaches is bacteriological quality monitoring of beach water quality.

Bacteriological monitoring programmes are necessary to identify faecal pollutants, their sources and occurrences, to develop preventive measures and also to assess the efficacy of corrective actions. The onus lies on the academic community to trigger such re-alignment of priorities by embarking on carefully planned surveillance studies that generate data on the bacteriological quality of recreational beaches. It is expected that data generated from such be disclosed to the public via various platforms that should include publishing in both local and international journals. With this public disclosure in place, the citizenry at large will be informed about the quality of available recreational beaches in the nation. It will also trigger behavioural patterns that need to be encouraged or changed to make the beaches a better place. Furthermore, the fear of a demeaning public perception of recreational beaches in country might trigger in other relevant stakeholders, the desired political will power for a change. It is however noteworthy to mention that at the initial stage of this process, unlimited public disclosure of such quality data may perceivably threaten an already built-up reputation in beach tourism, it will ultimately engender political will power for improvements. This wave of change ultimately reaches the industries as the government takes both proactive and reactive steps to protect and restore the quality of available recreational beaches through various policy instruments.

Another approach that could be founded on a robust bacteriological monitoring scheme is beach water profiling, an approach gaining increasing acceptance in Europe. A fundamental objective of this approach is to allow the citizenry to make an informed choice on which beach to choose for recreational activities. This is done via the annual public disclosure in the form of a report, the bacteriological quality of available coastal and freshwater bathing waters. The profiles include a general description of the bathing water, where wastewater outfalls and combined sewer overflows (CSOs) are positioned, potential sources of pollution, management measures, the location of the sampling point and how the beach has performed against bathing quality ratings over the previous years. A further improvement of this approach highlights designation of beach managers for each beach responsible for putting up signs at each designated bathing waters advising on the current water quality and potential pollution sources. The Department of Environment has made significant efforts in this direction especially for river water monitoring. Within a 20-year contract that began in 1995, Alam Sekitar Malaysia Sdn. Bhd. (ASMA) in partnership with DOE maintains a network of 1,063 manual sampling sites throughout Malaysia, as well as 15 continuous unattended monitoring stations at critical locations (YSI, 2007). The agency also routinely monitors under the classification of ‘marine water’, several samples from developmental islands, resort islands, marine park island and protected islands (DOE, 2006). Public beach waters appear left out in their schemes as data related to their quality are not made available in the public domain. It may be argued that focusing on quality monitoring rivers is a better approach especially for those that drain into coastal waters, for example, Sungai Redang. The possibility of having several direct sewage discharge into sea particularly with a booming coastal tourism industry clearly undermines this approach.

A robust bacteriological monitoring of beaches could form a basis for the introduction of benchmarking systems. However, it may amount to time-wasting efforts, canvassing for robust bacteriological monitoring schemes or benchmarking for beaches without the existence of bathing water quality standards. Currently, the only existing standard for beaches is the Interim Marine Water Quality Standards (IMWQS) which have been used as the benchmark for marine monitoring program in 1978 for Peninsular Malaysia and in 1985 for Sabah and Sarawak. The parameters measured are majorly *E. coli*, total suspended solids, pH, disoolved oxygen, levels of mecury, nitrate, nitrite, ammonia, cadmium, lead, arsenic and cupper. Studies conducted by EPA (EPA, 2011) to determine the correlation between different bacterial indicators and the occurrence of digestive system illness at swimming beaches suggest that the best indicators of health risk from recreational water contact in fresh water are *E. coli* (fecal coliform) and *Enterococci* (a subgroup within the fecal streptococcus group). For salt water, *Enterococci* are the best. A suggested approach for the currently existing Water Quality Index will be a revision that incorporates additional FIB parameters, a combination of total coliforms (TC), faecal coliforms (FC) and faecal streptococci (FS) as useful parameters for pollution indication. Beaches could then be classified as good, fair or poor based on counts obtained per 100ml for each of these three FIBs. It is noteworthy to assert that the government of Malaysia has made giant strides in this direction particularly for rivers in Malaysia. Water quality data from the 143 river basins throughout Malaysia are used to determine the water quality status weather in clean, slightly polluted or polluted category and to classify the rivers in Class I, II, III, IV or V based on Water Quality Index (WQI) and Interim National Water Quality Standards for Malaysia (INWQS) every year. It is hoped that a similar classification scheme be developed for recreational beach waters.

Certification schemes such as the Blue Flag could be encouraged for recreational beaches in Malaysia. The blue Flag is a voluntary environmental certification programme designed for beaches and marinas. It is an environmental education programme and incentive meant to improve the quality of water in coastal destinations. Usually, it is awarded to locations which have achieved the highest quality in water, facilities, safety, environmental education and management. In Jamaica, this certification program requires that water quality sampling be done once every fortnight for 2years prior to application; preparation and submission of application to the National Blue Flag Jury and further submission if approved, to the Blue Flag International Jury for review. Blue Flag is a prestigious, international award scheme which acts as a guarantee to tourists that a beach or marina they are visiting is one of the best in the world. The Programme has been in operation since 1987 within Europe Operating outside Europe since 2001. A total of 3012 beaches and 638 marinas (2010/2011 season) have Blue Flag certification worldwide in 41 countries across Europe, South Africa, Morocco, Tunisia, New Zealand, Brazil, Canada and the Caribbean. Within the currently available institutional framework, a similar program could be designed for beaches in Malaysia.

Another approach is that of capacity building. The aims of beach water quality monitoring programmes could be enlarged to accommodate community participation, partnership, ownership and willingness to take remedial actions if necessary. It can provide useful links between existing monitoring programmes and relevant communities to exchange useful information and ideas. Members of the academia could thus form stakeholder partnerships with available local and international NGOs to present scientific data on quality of recreational beaches in simple formats to the larger public and relevant enterprises. This approach develops the potential and ability of stakeholders to make and implement decisions that will lead to more sustainable beach usage, by increasing their level of awareness, understanding and knowledge. Although this approach is flexible, precise, securing long-term benefits; it can also be time consuming, requiring the use of skilled personnel (UNEP & UNWTO, 2005). Capacity building is important for beach water quality monitoring because often times, local communities act as polluters and or end receivers of pollution in recreational beaches. This thus places them in a vantage position to serve as eyes and ears of the authorities (Kailasam, 2007). Already existing local processes and programs such as the Malaysia Environment Week could be built upon to achieve this.

Having identified the potential of beach water bacteriological quality monitoring, its success is however hinged on political will power. Globally, water quality monitoring programmes, worldwide, are under severe stress as governments reduce budgets, downsize, and shift priorities (FAO, 1996). Yet, the need for reliable water quality information remains pressing. Given the opportunities that exist for new scientific research and need-based data collection designs, the academic can collaborate with the government to make management decision for the purpose of achieving the goal of sustainable beach management. With this working arrangement put in place, government will not see public disclosure of generated data, ideas or similar suggestions as a threat to existing structures but a platform that will engender the needed stakeholder collaboration between the academic community, the government and other relevant stakeholders. Technocrats, within and outside the academic community can work together with the government within budget realities to facilitate the introduction or revision of existing programs, legislation, policies, and economic instruments in order to promotes sustainable beach management. For instance, as suggested by the Institution of Malaysian Engineers, the Department for Environment (DOE) in conjunction with the National Water Services Commission/Suruhanjaya Perkhidmatan Air Negara (SPAN) could enforce environmental regulations for new constructions which should include water quality limits for effluents of at least Standard A as stipulated in the Environmental Quality Act, 1974 (IOE, 2011).

Also in this direction, economic instruments are important tools that can be adopted by the government to promote sustainable use of beaches. This may include imposition of taxes and charges as well as financial incentives and agreements. It is desired that such taxes and charges be carefully constructed in such a way that it goes beyond penalising unsustainable practices. It should engender the desired behavioural change in the polluting industries and citizenry at large. It is expected that such taxes will not be too low as to encourage polluters to prefer to keep polluting and paying the tokens fixed as charges. On the other hand, it must not be too high that it tends to alter the behaviour of consumers and enterprises, through their impact on prices, costs and income. Getting a desired balancing may however be challenging as they are indirect instruments with often unpredictable outcomes. Revenue generated from the application of these financial instruments may be redirected to academic community in collaboration with the Department of Environment for the purpose of funding more beach surveillance studies. Apart from collectible revenues, government may shift from the receiving end by delineating part of accrued revenue to provide fiscal incentives to actors deciding to change or incorporate environmentally friendly technologies.

Much has been discussed on the inertia bacteriological water quality monitoring could initiate for adaptable programs and policies aimed at sustainable beach management. It should be noted however that this paper does not present bacteriological water quality monitoring or data generation as a perfect panacea to the myriads of issues related to beach water quality management in Malaysia. The intricacies associated with study design, methodology and data collection in water quality monitoring schemes are well-documented (Ongley, 1993). Notable however in most of the published reports in Malaysia is an approach which seeks to answer specific questions vital to policy decision making. This need-based approach offer significant advantages over the data-driven approach which is still being used in most developed countries. On the contrary, even after many years of expensive data collection, these schemes have not been able to help such nations decipher whether available water resources are getting better or worse. According to Ongley (2000), this has resulted in significant shrinking down of conventional water quality data programs and realignment to a need-based approach. The challenge is thus no longer that of accessing information but one of integrating information in a systematic manner for the purpose of making decisive policy judgements on beach water quality management. Hopefully in the future, more studies in Malaysia will emerge with design criteria that pre-identify core management issues to be addressed. Whether they are data for public information, government policy and planning, regulatory purposes or for the purpose of general public health; other technical details associated with such proposed study will flow along this path.

## 5. Conclusion

On a final note, only few published reports exists on the bacteriological quality of recreational bathing water in Malaysia. Most of these are based on the use of conventional faecal indicator bacteria approach. Generally, these studies report varying levels of pollution with the most recent suggesting that the water available for bathing at the popular beach is not safe for recreational purposes. Partly due to the insufficient published need-based information in this regard, a major contributing factor to the current state of coastal beaches in Malaysia is that of an absence of political and stakeholder will power to set in motion action plans with a view to improving the quality of these beaches. The research community could help provide inertia by generating bacteriological data based on carefully planned experimental designs, for the purpose of supporting early warning systems and triggering public behavioural change and the political will power for high-level policy decision making on sustainable beach management. Beach water profiling, benchmarking, certification programs such as Blue Flag are direct approaches that could be built on such bacteriological monitoring schemes. Capacity building and networking with government agencies and other relevant stakeholders are indirect approaches that could also be explored.
